# Ventricular–Arterial Coupling in Takotsubo Syndrome: Controversies and Opportunities

**DOI:** 10.3390/jcdd13050175

**Published:** 2026-04-22

**Authors:** John E. Madias

**Affiliations:** 1 Icahn School of Medicine at Mount Sinai, New York, NY 10029, USA; madiasj@nychhc.org; Tel.: +1-(718)-334-5005; Fax: +1-(718)-334-5990; 2 Cardiology Department, Elmhurst Hospital Center, 79-01 Broadway, Elmhurst, NY 11373, USA

**Keywords:** Takotsubo syndrome, ventricular–arterial coupling in Takotsubo syndrome, left ventricular end-diastolic pressure in Takotsubo syndrome, systemic vascular resistance in Takotsubo syndrome

## Abstract

The present review focuses on the particulars of the hemodynamic assessment of patients with Takotsubo syndrome (TTS), i.e., blood pressure, cardiac output, systemic vascular resistance, left ventricular ejection fraction, left ventricular–arterial coupling (VAC), left ventricular end-diastolic pressure (LVEDP), and their importance in the management of patients with TTS, and the unraveling of its pathophysiology. In addition, the review discusses the discrepancies noted in the reported literature on the LVEDP and the left VAC, speculating as to the underlying reasons, and providing recommendations.

## 1. Introduction

Takotsubo syndrome (TTS), for which pathophysiology is still elusive, is a cardiac affliction often considered in isolation as a cardiac, instead of a cardiovascular (i.e., cardio-circulatory) illness. In this context, the associated systemic blood pressure (BP) is viewed as a consequence of the underlying blood volume and viscosity, cardiac output (CO), and systemic (aka peripheral) arterial wall vascular resistance (SVR), assuming no influence on them is exerted by the pathogenetic mechanisms of the ongoing TTS pathology which has affected the heart. Of course, this is wrong, since TTS is a cardiovascular, and even systemic (more than vascular), whole-body malady [1], not exclusively exerting a nosogenic effect only on the heart via a brain–heart connection [2], but also resulting in a cerebral, pulmonary, gastrointestinal, renal, peripheral, systemic, and pulmonary, vascular, “total body TTS” affliction, i.e., involving all body organs and organ systems. On contemplating this notion, one should make a distinction between body organ/systems morbidity due to left ventricular (LV) and right ventricular (RV) decompensation (i.e., secondary), resulting in poor arterial organ perfusion or passive venous congestion, and the results of a direct effect on organs/systems from whatever causes TTS (i.e., primary) (e.g., autonomic sympathetic nervous system [ASNS] surge, catecholamine outpouring, local microvascular/endothelial dysfunction, arterial/microvascular spasm, metabolic derangements, etc.). Accordingly, and as an example, in patients with renal failure, requiring renal replacement therapy in the setting of TTS [3,4], one should differentiate between the “prerenal” influences of the cardiac TTS due to consequent heart failure (HF) or cardiogenic shock (CS), and the associated “local” primary renal TTS derangements. Patients with TTS with chronic renal failure had a higher rate of acute renal injury and admission creatinine, and this biomarker was associated with lower left ventricular ejection fraction (LVEF) on admission, and higher rates of CS, in-hospital bleeding, other major adverse cardiovascular events, and all-cause mortality during a 5-year follow-up [5]. Similarly, there are examples of a “local” gastrointestinal TTS affliction in patients with gastrointestinal symptoms and signs, which could be viewed as the triggers for TTS; but, they could also be due to an independent gastrointestinal TTS component [6,7], to the point that it occasionally brings to mind the proverbial “chicken or the egg” causality dilemma. Similar considerations have caused some to speak of a “cerebral TTS” in patients with TTS triggered by transient global amnesia [8], which, although could be viewed as a neurological/psychiatric affliction which could trigger cardiac TTS, it has itself been triggered by the same ASNS/catecholamine surge, which has led to the cardiac TTS. Another example of “noncardiac” accompaniment of TTS is the neurogenic pulmonary edema associated with aneurysmal subarachnoid hemorrhage, which is not necessarily a consequence of cardiac deterioration from TTS, but is attributed to multiple ASNS-/catecholamine-induced derangements (i.e., vasoconstriction, alterations in endothelial function, increased vascular permeability, etc.) directed to the lungs [9]. A case in point is of a 61-year-old patient who suffered a right-sided stroke associated with TTS pulmonary edema, as well as hepatic impairment, with the authors attributing the lung and liver complications, in agreement with this author, to “severe hypoxemia which may also be modulated by neurohumoral mechanisms following massive cerebral infarction” [10]. One wonders whether many clinical afflictions, associated with emotional or physical stresses, and involving many body organs/systems, are accompanied by undetected subclinical or mild forms of TTS [11] and thus rendering TTS highly underdiagnosed, since many affected patients are probably often not evaluated with electrocardiography (ECG) or transthoracic echocardiography (ECHO). Finally, a vascular “TTS”, which is the focus of this commentary, must be present in patients with TTS to explain the observed hypotension, low SVR, preserved CO, markedly reduced LVEF, and low/normal LV end-diastolic pressure (LVEDP) [12,13,14,15,16], bespeaking of an altered ventricular–arterial coupling (VAC). Involvement of other body organs/systems, including the systemic arterial and the pulmonary vasculature in patients with TTS, should also be expected, if one expects the nosogenic effects of an unbridled ASNS and/or outpouring of catecholamines to influence the entire body, rather than only the heart. The notion that TTS is indeed a cardiovascular, and not a cardiac, “total body” affliction, is in keeping with the ideas of Hans Selye, the Father of the “stress field”, who provided laboratory and clinical proof that stress of all different varieties (i.e., somatic or emotional [negative or positive]), has a noxious effect on many body organs/systems, since the stress induced is nonspecific in its consequence (i.e., stereotyped in its manifestations), and independent of the specific characteristics of the triggering stressor [17,18]. The occasionally noxious stressful effects encountered with positive emotions, described originally by Selye, are echoed by the occurrence of the “happy heart syndrome” as a sub-phenotype of TTS [19]. It should be admitted herein that the reference to the “total body TTS” and the organ-specific “local” manifestations concepts, directly instigated by ASNS overstimulation and/or outpouring of catecholamines, is more assertive than what the laboratory and clinical data really allow, since very little has been clearly established and thus much is speculative or inferential, indeed reflecting an extrapolation of Hans Selye’s ideas on the stress syndrome affecting the same multiple body organs and systems, irrespective of the nature of the stressful triggers.

## 2. VAC in TTS

In the context of hemodynamic assessment of cardiac patients, clinical practice and research reports usually make reference to the CO, BP, heart rate (HR), SVR, and LVEF, usually provided by Doppler/ECHO and transpulmonary thermodilution testing [20]. CO, stroke volume (i.e., CO/HR), and SVR (i.e., mean arterial BP—right atrial (RA) [or central venous pressure]/CO) [20], are also referred to, and are also indexed to the body surface area of the individual patient. The LVEDP, another metric of hemodynamics, is less often reported than previously, since LV contrast angiography (LVAng), for which a pigtail catheter needs to be inserted into the LV, does not always follow coronary angiography (CANG) in patients with TTS when such a procedure is delayed, and when the LV function has already been evaluated by an earlier ECHO. When the LVEDP is measured, details should be provided about the time of such measurement, and whether it was preceded by CANG and/or LVAngio, since there has been evidence that angiographic contrast media injections increase the LVEDP, and that such alterations may be predictive of underlying HF [21]. On occasion, when catheterization’s indication is other than for the evaluation of suspected/proven acute coronary syndromes (ACS), RV catheterization is carried out, which also includes the measurement of pulmonary capillary wedge pressure (PCWP) which only partially reflects LVEDP, and thus can be employed as a surrogate of LVEDP, with many qualifications needed to be taken into consideration [22]. The complex relationship among CO, BP, HR, LVEF, SVR, and LVEDP is manifest as the LV-VAC, which expresses the existing dynamic relationship of the LV contractility to the systemic arterial load (i.e., representing a global assessment of cardiovascular efficiency), in health and disease. LV-VAC is defined as the ratio of arterial elastance (EA) (i.e., load or a measure of afterload) to the LV end-systolic elastance (ESE) (i.e., contractility), with normal coupling being ~1.0, with both ratios expressed in mmHg/mL, and with both EA and ESE estimated in the ECHO laboratory [23]. Of interest is that patients, with CS due to ACS or decompensated HF [20], were documented to have a range of SVR, from low (similar to the ones noted in septic shock) to high, suggesting that different patients may require fluid administration or diuresis, vasopressors (e.g., norepinephrine, vasopressin, phenylephrine), or inotropes (i.e., dobutamine, milrinone, isoproterenol). Hemodynamic assessment of the pulmonary circulation is also made by reference to the RA and RV systolic/diastolic/mean pressures, systolic/mean/diastolic pulmonary pressures, PCWP, and pulmonary vascular resistance (i.e., mean pulmonary artery pressure—left atrial (LA) pressure [or PCWP]/CO), based on right heart catheterization, and currently via ECHO testing; indeed, ECHO/Doppler can be implemented in the serial estimation of LA pressure, using transmittal and tissue Doppler imaging, and for RA pressure, using the vena cava respiratory dynamics [24]. RV VAC can be assessed by two-dimensional ECHO strain; RV pulmonary artery coupling can be measured as the ratio of either tricuspid annular plane systolic excursion (i.e., TAPSE) and RV global longitudinal strain, or RV free wall strain to pulmonary artery systolic pressure, and abnormalities in these metrics were associated with cardiovascular complications and, when employed, they superseded the prognostic reliance only on LV LVEF alone [25]. The assessment of VAC parameters, which traditionally are defined as the combined markers of arterial and myocardial function, and all the other markers mentioned in this review which characterize the cardiac pumping efficiency and the adequacy of the peripheral vasculature in supplying peripheral organs’ perfusion, rely on high quality LVAgio and, preferably, the employment of a high-fidelity micromanometer-tipped LV catheter [15] for the calculations of volume pressure loops during the entire cardiac cycle; the same applies to ECHO/Doppler-based calculations, which are feasible only with studies of the highest quality in terms of chamber volumes, and velocity of intracardiac and vascular jets’ measurements. The necessary specific measurements and mathematical formulas for the acquisitions of these cardiovascular markers are available in the literature, with minor variation among authors. What is more important for the readers is to contemplate that what is currently done in practice and what is reported in most of the literature, is not enough for the adequate hemodynamic characterization of our patients with TTS, particularly in reference to the time-varying VAC. In reference to the latter, one should understand that what is provided by the one-time LVAngio assessment is not adequate, and that serial hemodynamic characterization should rely on serial ECHO/Doppler testing. Two studies, nine years apart [15,26,27], have provided sophisticated evaluation of hemodynamics, the first within the first 48 h, and the second within 24 h after the patients’ hospital admission. The studies required continuous LVAngio-based volumetric and LV blood pressure assessments. The first [15] revealed in women with TTS similar findings with women after a left anterior descending coronary artery occlusion anterior AMI, both with differences with the studied controls, characterized by increased diastolic volumes, pressures, and stiffness (i.e., the reciprocal of diastolic compliance, employing a physical tissue characterization term), lower LVEF (with patients with TTS having lower LVEF than patients with AMI), lower stroke work, decreased contractility, and abnormal LV VAC, in spite of an assumed different pathophysiology. The other study [26,27] compared mainly women (only one man was included) with TTS with controls, employed LV pressure–volume loops, and found impaired contractility (i.e., decreased end-systolic elastance), maximal rate of change in systolic pressure over time, end-systolic volume at a pressure of 150 mm Hg, shortened systolic period, a pressure–volume diagram with a rightward shift in the pressure–volume relationship, increased LV end-diastolic and end-systolic volumes, preserved LV stroke volumes in spite of the lower LVEF, prolonged active relaxation, minimal rate of change in diastolic pressure, reduced mechanical efficiency, reduced stroke work, and a similar diastolic passive stiffness and total pressure–volume area compared with that of controls, suggestive of decreased phosphorylation of myofilament proteins, which aligns with the recommendation of some to use levosimendan in patients with TTS and HF and/or CS. Although such sophisticated assessments, as described above, are necessary to characterize the hemodynamic profile of patients with TTS, they require invasive means, can by carried out only once, and thus cannot evaluate patients serially, which is important in a temporally changing malady, and were accomplished without provided information on the time interval between the inception of the TTS episodes and the performance of such investigations.

Another matter that needs special attention is the association of the emergence of TTS with left ventricular outflow tract obstruction (LVOTO); there is variation in the literature reports about the frequency of LVOTO in patients admitted with TTS. LVOTO, in the setting of TTS, is associated with mitral valve (MV) regurgitation, hypotension, HF, and CS, and requires special management focusing on the avoidance or discontinuation of inotropes, including catecholamines, careful administration of fluids, introduction of intravenous β-blockers, preferably of the short acting (e.g., esmolol) or ultrashort-acting (e.g., landiolol) variety, and avoidance of intra-aortic balloon pumping (IABP), when LV assist devices are called for [28,29,30]. It is possible that LVOTO is more frequent than currently thought, and may be transiently present early in the emergence of TTS, with some even postulating its pathogenetic role in TTS [28,29]. During individualized assessment for a TTS patient, identifying the presence or absence of concomitant LVOTO should arguably be the first step in the comprehensive hemodynamic evaluation, followed by an assessment of other features. This aspect might benefit from earlier and more explicit emphasis; accordingly, this author [28,29,30] has repeatedly emphasized the importance of frequent serial point-of-care ECHO, employed by many members of the caring team to detect LVOTO and/or MV regurgitation, in order to prompt or expedite referral for a formal high-quality ECHO acquisition. Given this review’s focus on LV VAC, LVOTO in the setting of CS deserves more explicit attention. Recent evidence suggests that the development or worsening of LVOTO directly attributable to inotropic or vasoactive support may be less frequent than often assumed [31]. This is clinically important because, although β-blockers are pathophysiologically preferred therapies for TTS in general, and associated LVOTO in particular, considering β-blockers’ side effects of hypotension, worsening of HF, and bradycardia, we may reconcile with the therapeutic approach to proceed with slow vasoactive titration or a careful alternation between therapies that are not perfectly aligned with the theoretical model of using strictly β-blockers. However, the short half-life of these agents (vide supra) provide some reassurance for their employment. Recent limited evidence suggests that Impella may be a potentially attractive option in TTS complicated by LVOTO and CS [32], considering the differences in the physiological effects among circulatory support devices; accordingly, while IABP improves CO and reduces afterload, which potentially may worsen the intraventricular gradient and the veno-arterial extracorporeal membrane oxygenator (V-A-ECMO) may have competing effects on loading conditions, Impella may be particularly appealing because it unloads the LV in a manner consistent with the pathophysiologic framework of the emergence and worsening of LVOTO in the setting of TTS.

There has been interesting works about a diagnostic overlap among true hypertrophic cardiomyopathy (HCM), segmental LV septal hypertrophy, not necessarily of the HCM autosomal dominant type, and structural abnormalities of the MV leaflets and corda tendineae, associated with apical ballooning which may or may not represent TTS, with some of these patients revealing persistent LVOTO following recovery of an acute episode associated with a hospital admission [33,34]. Because of the above, LV VAC in patients with TTS and LVOTO may represent a complex, dynamic, and difficult to interpret and categorize hemodynamic variant, totally different from the one encountered in TTS patients with apical ballooning but without LVOTO. Instrumental in the differentiation between true HCM and “septal pseudohypertrophy”, due to segmental myocardial edema masquerading as HCM, is the early and follow-up cardiac magnetic resonance imaging testing, which in the case of TTS associated with “septal pseudohypertrophy”, will show that increased septal thickness has dissipated [35].

When evaluating an individual patient with TTS in terms of his/her initial and longitudinal hemodynamic state, for the choice of fluid volume administration or diuresis, the use of vasodilators, vasopressors, inotropes, implementation of LV and/or RV assist devices (i.e., IABP, LV assist devices [e.g., Impella], or VA-ECMO), and the subsequent clinical response to these measures, one should contemplate the following: (1) Normal LV VAC is characterized by an equipoise of optimal LV diastolic filling and LVEDP and arterial load resulting in an adequate CO and BP. (2) In some TTS patients, the LV-VAC is appropriately maintained in spite of the reduced LV contractility, expressed by a relatively normal afterload, BP and CO. (3) In some other TTS patients, the LV-VAC is impaired, with the reduced LV contractility associated with reduced CO, increased LVEDP, and low BP, with partial compensation provided by the Frank–Starling mechanism, exemplified by the increased LVEDP and higher LV preload/volume, a picture not unlike the one encountered in ACS, AMI, HF, or CS. (4) During the clinical course of TTS, patients are expected to move from one to the other hemodynamic phenotypes described above, as a result of the clinical trajectory, due to the TTS spontaneous healing process being underway, or by following administered therapies. (5) While related, LV-VAC and SVR are different hemodynamic parameters, the former referring to a ratio representing the efficiency of the heart’s function (i.e., contractility [i.e., ability of the cardiac muscle to shorten/produce force] and compliance or stiffness [i.e., resistance of the cardiac muscle to being lengthened/stretched]) in confronting the arterial load, while the latter reveals the pressure/flow against which the heart pumps. (6) Estimating and thinking about the LV-VAC in TTS often leads to counterintuitive conclusions about diagnosis and management; unlike HF CS, ACS, and AMI, issues are more complex, because in TTS, we are often dealing with two-compartment LV models with dyskinetic/akinetic/hypokinetic vs. normokinetic/hyperkinetic LV regions, with or without LVOTO, which further complicates matters; one could also contemplate a three-compartment LV model, in the case of mid-ventricular TTS morphological variants, with one dyskinetic/akinetic mid-ventricular and two (i.e., apical and basal) normokinetic/hyperkinetic compartments. (7) Although the hemodynamic parameters discussed above are connected with each other via mathematical formulas, appearing sometimes intimidating, we should keep in mind that LVEDP or PCWP, LV diastolic volumes, and LVEF on one hand, and CO and BP on the other hand, with their varying relationships, express the LV efficiency in pumping an adequate blood volume for peripheral organ perfusion, with the caveat that patients with TTS may show during at least at some portion of their clinical course counterintuitive behavior in these two opposing sets of parameters, further complicated by the fact that different LV segments display different mechanical efficiencies.

## 3. VAC Discordances in the TTS Literature

There is proof that there are disturbances in the VAC in patients with TTS, in the sense that this metric is different in such patients than the one encountered in normal controls, and in patients with ACS and AMI, concomitantly evaluated [14]; accordingly, in the experience from the Sahlgrenska University Hospital of Sweden, left ventricular filling pressures were normal or near normal, and there was a decreased SVR and preserved CO in patients with TTS, in contrast to the patients with AMI, who had elevated SVR and decreased CO, while the LVEF was worse in patients with TTS than the one encountered in AMI. This altered VAC ensures adequate peripheral perfusion in patients with TTS (16), which probably relates to systemic vasodilation engendered by a decreased sympathetic tone, a counterintuitive finding for a condition allegedly due to an increased ASNS activity and a catecholamine surge, which is expected to produce systemic arterial vasoconstriction. Indeed, the outpouring of catecholamines, with their expectant stimulation of vascular α_1_-receptors, is expected to result in vasoconstriction and an increase in the SVR. However, the observed hypotension, preserved CO, and low SVR in patients may reflect a sympathetic adrenergic system-derived vasodilation imparted by the stimulation of β_2_-adrenergic receptors, resulting from epinephrine outpouring; such a notion may be a parallel to the findings in a rat experimental model of TTS, imparted by an intravenous epinephrine administration, and attributed to a switching of epinephrine signaling through the pleiotropic β_2_-adrenergic receptors from the canonical stimulatory G-protein-activated pathway to the inhibitory G-protein-activated cardiodepressant type [36]. However, these authors did not elaborate on the hemodynamics, resulting from the intravenous epinephrine administration in their TTS rat model. Additionally, TTS induced in patients in the setting of pheochromocytoma, which is due to episodic massive outpouring of catecholamines from an adrenal tumor, is often associated with episodes of profound hypotension interspersed with phases of peripheral vasoconstriction and severe hypertension; indeed, a recent review on the topic reported that >30% of the patients with pheochromocytoma and TTS presented with hypotension, while the remainder presented with hypertension [37]. Of interest is that the patients who presented with hypotension had a higher proportion of the typical apical variant of TTS, which may imply that epinephrine, rather than norepinephrine, was the offender, in parallel with the above cited study [36], although the authors of that review [37] did not differentiate between epinephrine vs. norepinephrine and their metabolites but reported merely on elevated catecholamines. There is variation in the type of catecholamines found to be elevated in the setting of TTS [38], and it is conceivable that the type of specific catecholamine (i.e., epinephrine, norepinephrine, or dopamine) found to be elevated in patients with TTS may have an association with the TTS morphological variant (i.e., apical, mid-ventricular, reverse), or typical (i.e., apical) or atypical (i.e., non-apical) [39], and the underlying VAC, either hypertension or hypotension. In carefully carried out experiments, it has been shown that LVEDP was lower in rats with TTS as compared with rats with AMI, and this finding was mirrored by the lower LVEF in patients with TTS, leading the authors to comment that hemodynamics are more favorable in TTS than AMI, with both rats and patients with TTS revealing a better compensated circulatory state [40]. The same group of investigators have recently reported that, although TTS patients have a “more extensive cardiac dysfunction” than patients with ST-elevation AMI, they have “milder symptoms and a better hemodynamic profile” [41], keeping with their previous animal and clinical investigations [12,14,16,39,40], but they documented similar LV-VAC, as assessed by Doppler/ECHO-derived SVR [42,43]. Some feel that the varied and complex hemodynamic phenotypes seen in TTS seem to relate more strongly to the presence or absence of physical stressors such as sepsis, severe cerebrovascular accident, and pheochromocytoma, or extracardiac comorbidities. In TTS cases without clear stressors or with purely emotional triggers, an elevated LVEDP appears more often in clinical practice, while abnormalities in SVR are less frequently observed. However, the latter is contradicted by the experience of the Sahlgrenska group (vide supra) investigators, who have found subnormal SVR, lower peripheral sympathetic activity, preserved CO during the acute phase (i.e., within 48 h after admission), and normal/mildly elevated LVEDP (i.e., “normal or near normal filling pressure”, as per the Gothenburg TTS criteria [14]) in their patients with TTS in comparison with the age- and gender-matched healthy volunteers, suggesting that TTS is a “cardio-circulatory syndrome in which cardiac dysfunction is accompanied by altered circulatory physiology” [14,16]. It should be noted, however, that these authors did not compare non-invasively assessed hemodynamics of their TTS patients according to the precipitating triggers, and their experience was limited to 12 patients. Indeed, the non-specificity of the stress response [17,18] suggests that the diversity of clinical severity phenotypes may be due to the interplay of TTS and other acute comorbidities [44]. Considering the convoluted variegated clinical trajectory of patients with TTS, and variation in the time interval between onset of illness, the eventual diagnosis of TTS, and the associated acute comorbidities [44], the issue appears complex, needing serious thought and careful scrutiny. This is imperative, since the Sahlgrenska group, in a recent report [41] that generated some debate [42,43], unlike their previous experience [14,16], found that women with TTS and AMI had similar longitudinal and global radial ECHO strain, SVR, and systemic vascular compliance (i.e., stroke volume/pulse pressure, another metric influencing afterload), and a lower LVEF.

## 4. Catecholamines and VAC

Although the pathophysiology of TTS is still elusive, the ASNS overstimulation and the outpouring of catecholamines as pathophysiological triggers are currently favored [14,28,29,36,38,39,40,45]. There must be some relevance to the types of catecholamines oversecreted and the VAC, since different catecholamine predominance is associated with different TTS morphological variants [39,40], and thus possibly different VAC abnormalities. While norepinephrine is considered as exerting its effect via overstimulation of the β_1_-adrenergic receptors, releasing noradrenaline, resulting in cardiomyocyte toxicity, adrenaline secreted mainly by the adrenal glands produces its noxious effects by overstimulation of the β_2_-adrenergic receptors, thus converting the positive to negative effects on cardiac contractility [36], manifesting primarily in the apical cardiac region which is endowed with the larger number of β_2_-adrenergic receptors. The explanation provided herein about the role of an intense β_2_-adrenergic stimulation and an apical susceptibility is supported by some animal laboratory work [36], but there is no evidence actually available revealing a pathogenetic mechanism of such a β_2_-adrenergic overstimulation primarily causing the apical TTS morphological variant in the human; accordingly, this remains a plausible mechanistic hypothesis, and not something that has been firmly established in the clinical setting. Thus, both norepinephrine and epinephrine, and possibly other catecholamines (e.g., dopamine) and other neuropeptides [45], may be at the roots of the LV and RV segmental hypocontractility. Undoubtedly, catecholamine blood sampling and its trend may be of value to ascertain the clinical stage of an evolving TTS episode, the association with VAC, and the deciphering of the pathophysiology of TTS.

## 5. LVEDP in TTS

Surprisingly, there is a large range of values of LVEDP reported in the literature, with some reporting low or normal LVEDP [12,14,16,39,40], while others reported high LVEDP [15,45,46,47,48,49,50,51,52], similar to the one seen in the ST-elevation anterior AMI with HF and CS, while others expressed puzzlement about the discrepancies [53]. Furthermore, granular patient data from the TTS World literature and the large range of LVEDP data values in reports without granular data, revealed a large number of patients with TTS and low/normal LVEDP [40] and PCWP values [13].

## 6. Diagnostics of VAC in TTS

Hemodynamic assessment, including LV VAC, of patients with TTS, requires measurements of BP (i.e., SBP, mean BP, and DBP), HR, LVEDP, PCWP, CO (and cardiac index), and RA (or central venous) pressure. While evaluation of certain parameters like LVEDP, PCWP, CO, and RA pressure requires invasive approaches (i.e., LV and RV, RA catheterization) [15] and provides only single measurements, all the above can be measured (or estimated) with Doppler/ECHO and thoracic bioimpedance technologies [20,24,54], thus providing serial measurements during the clinical course of patients (i.e., worsening or improvement), and comparisons of parameters before and after therapeutic interventions.

## 7. Improvements Towards Measuring/Reporting Hemodynamics in TTS

Discrepancies in the literature reporting on LVEDP, SVR, LV VAC, and other hemodynamic parameters may be due to a range of problems, stemming from the different timing of measurements in connection with the inception of the illness, variations in the LVEF and extent of LV/RV wall motion abnormalities, delays in making the diagnosis of TTS, and administration of inappropriate therapies, resulting from a late diagnosis of TTS. Serial timed measurements of BP, HR, CO, SVR, LV VAC, and LVEF, via Doppler/ECHO, or other non-invasive means, are in order (Table 1); such measurements have been provided only by the targeted TTS literature [15,26,27,43], but it is recommended that they be obtained serially in all patients. Also, such measurements should be considered in the context of symptoms, signs of cardiac decompensation, HF, or CS, and ECG evidence of bradycardia, tachycardia, atrioventricular blocks, or supraventricular and ventricular arrhythmias. It is imperative that reports of hemodynamic parameters in patients with TTS be made with reference to the timing of their measurements, and their association with the onset of the illness, stage of the clinical course, and results of other laboratory and imaging testing. Accumulated knowledge on the above will be helpful in the management of patients and will aid in the elucidation of the pathophysiology of TTS, particularly in reference to whether TTS is different than ACS and AMI. In perusing the massive literature on TTS, one becomes cognizant that there are many hemodynamic phenotypes with differing, ever changing LV and RV VAC profiles (Table 2), and thus, it follows that management should take into consideration the specific phenotypic expression the patient shows at the time of contemplated specific pharmacological and/or device-based therapies. Accordingly, we should also be quick in terminating chosen therapies when a patient appears to be converting to another hemodynamic phenotype, or plainly does not show improvement, or is deteriorating (e.g., the patient’s hemodynamic response to β-blockers, IABP, or catecholamine inotropes or vasopressors). The “therapy” column in Table 2 may give the impression of a management algorithm, but it should be considered that its wording is stronger than what the current evidence base actually supports; indeed, that part of Table 2 should be interpreted as a practical conceptual framework, and not as a prescriptive treatment pathway. Much more work is needed to provide us with therapeutic principles of proven value for the management of TTS.

## 8. Conclusions

Hemodynamic parameters, like BP, CO, LV VAC, SVR, and LVEF, among others, are useful in the management of patients with TTS and may contribute in the future to the unraveling of its pathophysiology. The review also discusses the discrepancies in the literature’s reported data regarding LVEDP and LV VAC, the possible underlying reasons for such variations, and what is needed to improve reporting on the hemodynamic state of patients with TTS.

## Figures and Tables

**Table 1:** Hemodynamic assessment of patients with TTS.

Practice and Most Literature	Targeted Literature	Future Recommendations
Blood pressure	Blood pressure	Serial blood pressure
Heart rate	Heart rate	Serial heart rate
	Cardiac output	Serial cardiac output
	Stroke volume	Serial stroke volume
	SVR	Serial SVR
	SVC	Serial SVC
LVEF	LVEF	Serial LVEF
LV-RWMA (Qual.)	LV-RWMA (Qual.)	Serial LV-RWMA (Qual.)
LV-RWMA (Quan.)	LV-RWMA (Quan.)	Serial LV-RWMA (Quan.)
BNP	BNP	Serial BNP

Abbreviations and acronyms: BNP = brain natriuretic peptide; LV = left ventricle; LVEF = left ventricular ejection fraction; RWMA = regional wall motion abnormalities; SVC = systemic vascular compliance; Qual. = qualitative; Quan. = quantitative; SVR = systemic vascular resistance.

**Table 2:** Hemodynamic phenotypes in patients with TTS.

Phenotypes	Diagnosis	Therapy
Minor symptoms/no complications	BP/HR/LVEF/CO/ RWMA/SVR	Fluid infusion/diuretics/ β-blockers, as needed
Hypertension and/or tachycardia	BP/HR/LVEF/CO/ RWMA/SVR	β-blockers
HF/normal BP	BP/HR/LVEF/CO/ RWMA/SVR	Diuretics; if no response: levosimendan/milrinone
HF/low BP or CS	BP/HR/LVEF/CO/ RWMA/SVR	Levosimendan/milrinone; if pulmonary congestion: diuretics/catecholamine inotropes & vasopressors while transitioning to Impella/IABP/ VA-ECMO
LVOTO without HF or CS	BP/HR/LVEF/CO/ RWMA/SVR	If low BP: fluid infusion/ phenylephrine/ β-blockers
LVOTO with HF or CS	BP/HR/LVEF/CO/ RWMA/SVR	While moving to Impella/ VA-ECMO, a trial of β-blockers/fluid infusion/ phenylephrine
Hemodynamic assessment should be carried out serially, except in the asymptomatic patients, although even such patients should be monitored carefully; we should be cognizant of the fact that patients with TTS can move abruptly from one phenotype to another, either improving or deteriorating.		

Abbreviations and acronyms: BP = blood pressure; CO = cardiac output; CS = cardiogenic shock; HF = heart failure; HR = heart rate; IABP = intra-aortic balloon pump; LVEF = left ventricular ejection fraction; LVOTO = left ventricular outflow tract obstruction; RWMA = regional wall motion abnormalities; SVR = systemic vascular resistance; VA-ECMO = veno-arterial extracorporeal membrane oxygenator.

## Data Availability

No new data were created or analyzed in this study. Data sharing is not applicable.

## Abbreviations

ACS	Acute coronary syndromes
AMI	Acute myocardial infarction
ASNS	Autonomic sympathetic nervous system
BP	Blood pressure
CANG	Coronary angiography
CO	Cardiac output
CS	Cardiogenic shock
ECG	Electrocardiography
EA	Arterial elastance
ESE	Left ventricular end-systolic elastance
ECHO	Transthoracic echocardiography
HCM	Hypertrophic cardiomyopathy
HF	Heart failure
HR	Heart rate
IABP	Intra-aortic balloon pump
LA	Left atrium
LV	Left ventricle
LVAng	Left ventricular contrast angiography
LVEDP	Left ventricular end-diastolic pressure
LVEF	Left ventricular ejection fraction
LVOTO	Left ventricular outflow tract obstruction
MV	Mitral valve
PCWP	Pulmonary capillary wedge pressure
RA	Right atrium
RV	Right ventricle
SVR	Systemic vascular resistance
TTS	Takotsubo syndrome
VAC	Ventricular–arterial coupling
VA-ECMO	Veno-arterial extracorporeal membrane oxygenator
